# Impact of different numbers of microsatellite markers on population genetic results using SLAF-seq data for *Rhododendron* species

**DOI:** 10.1038/s41598-021-87945-x

**Published:** 2021-04-21

**Authors:** Huaying Wang, Baiming Yang, Huan Wang, Hongxing Xiao

**Affiliations:** Key Laboratory of Molecular Epigenetics of Ministry of Education, Northeast Normal University, Changchun, 130024 China

**Keywords:** Genetics, Plant sciences

## Abstract

Microsatellites (simple sequence repeats, SSRs) are co-dominant nuclear markers that are widely used in population genetic studies. Population genetic parameters from different studies might be significantly influenced by differences in marker number. In our study, 265 sequences with polymorphic microsatellites were obtained from SLAF-seq data. Then, subpopulations containing different numbers (5, 6, 7,…, 15, 20, 25, 30, 35, 40) of markers were genotyped 10 times to investigate the impact of marker numbers on population genetic diversity results. Our results show that genotyping with less than 11 or 12 microsatellite markers lead to significant deviations in the population genetic diversity or genetic structure results. In order to provide markers for population genetic and conservation studies for *Rhododendron*, 26 SSR primers were designed and validated in three species.

## Introduction

Microsatellites (simple sequence repeats, SSRs) have been the most frequently used genetic marker in population genetics over the past 20 years^1^. Although single nucleotide polymorphic loci (SNPs) have been used in a variety of genetic studies^2^ recently, they still have not replaced SSRs completely as microsatellites are highly informative, codominant, exhibit high specificity, are transferable among related species and have relatively low costs. As such, microsatellites continue to be widely used, especially for wild species, particularly when sample sizes are large. The number of loci used for genotyping is one of the key issues concerning the use of microsatellites. Koskinen et al. show a substantial decrease in standard deviation estimates of the interpopulation genetic distances by increasing the number of loci from six to seventeen. They indicate that the stability of commonly used genetic distances and phylograms is determined by the number of microsatellites investigated^3^. In addition, the results obtained from 205 red deer indicate significant effects on population genetic parameter if the number of microsatellite loci smaller than six^4^. However, in experimental studies of *Drosophila nigrosparsa*, Arthofer et al. demonstrate that a large proportion of individuals can still be correctly assigned to population of origin when using eight loci, and the population structure is still retained when using only two highly polymorphic loci^5^.

Unfortunately, to investigate the impact of different numbers of loci on population genetic results, the subpopulations containing different loci were generated from the total set of 16 or 17 microsatellite loci in previous studies^3,4^. Compared with the total number of microsatellites in genome, 16 or 17 microsatellite loci are too low and may be inconsistent with actual experimental design. This is limited by previous de novo SSR development, which can be a tedious and costly process^6^. More recently, next generation sequencing (NGS) technology has facilitated the development of hundreds microsatellite loci based on sequence data with reduced cost and effort^7–10^, offering the possibility of resampling different numbers of microsatellites from genome.

In our previous study, we estimated the population genetic structure and demographic history of two closely related species of *Rhododendron*, *R. dauricum*, and *R. mucronulatum*, distributed in northeastern China, using 664,406 SNPs based on specific-locus amplified fragment sequencing (SLAF-seq), a recently developed, high-resolution strategy for the discovery of large-scale de novo genotyping of SNPs^11,12^.

Here, using SSR sequences from this dataset, we evaluated the impact of using different numbers of SSR loci, generated from the whole genome, on the stability of population genetic results. Furthermore, we used the SLAF-seq data to develop polymorphic microsatellite markers for *R. dauricum* and *R. mucronulatum*. *Rhododendron* species are widely distributed around the world ranging from tropical to polar climates, and used as valuable horticultural plants due to their beautiful vegetative forms and remarkably bright-colored flowers^13^. The microsatellite markers developed in this study will aid genetic diversity studies of *Rhododendron*.

## Materials and methods

### SLAF data and microsatellite mining

On the basis of our previous SLAF sequencing data of *R. dauricum* and *R. mucronulatum* (accession number: PRJNA589346), we removed some populations that contain only one individual and merged some populations were close to each other geographically. Our dataset for this part consisted of 38 *R. dauricum* and 25 *R. mucronulatum* samples, and the sample vouchers have been deposited at the Northeast Normal University Herbarium (NENU, Table S1). All samples were identified by an expert taxonomist, Dr. Mingzhou Sun and Prof. Hongxing Xiao, Northeast Normal University, China. All filtered SLAF reads were clustered by the BLAT software according to sequence similarity to create SLAF tag sequences^14^. Polymorphic SLAF tags showed sequence polymorphisms between different samples.

Microsatellite motifs from di-nucleotides to hexa-nucleotides were identified from the polymorphic SLAF tag sequences in MISA-web (http://misaweb.ipk-gatersleben.de/). The lowest threshold of repeats for dinucleotides was set to six, while all others were set to five. Two SSRs motifs with the maximum interruption less than 100 bp were considered as one compound microsatellite.

Additionally, insertions/deletions (INDELs) were called using the program SAMtools ^15^ and Genome Analysis Toolkit (GATK)^16^ with our previously used parameters^11^. Raw INDELs were filtered using our custom Perl scripts with the cutoff “mapping quality (MQ) > 30, read depth (DP) > 3.” Moreover, PLINK 2^17^ was used to further filter with the minor allele frequency (MAF) of 0.04 and maximum missing rate of 0.1. Finally, microsatellite genotypes for each individual were determined based on the sequence length of core motifs.

### Genetic diversity and structure based on different number of microsatellite loci

Inbreeding coefficients (FIS) and corresponding *p*-values, which indicate whether markers or populations deviate from Hardy–Weinberg equilibrium, were tested by 1000 random permutations using FSTAT version 2.9.3.2^18^. When FIS values for a locus deviated significantly from zero (p < 0.01), loci were excluded from further analyses. In addition, number of alleles (NA), allelic richness (Ar) and genetic diversity (Hs)^19^ were calculated for each species using FSTAT software. The population genetic structure was analyzed using the Bayesian clustering program STRUCTURE version 2.3.3^20^. The admixture model with correlated allele frequencies was chosen, as recommended for faint population structures. The number of clusters (K) assumed was set to [1, 10], and each value of K was run 10 times. Each run was performed with 20,000 MCMC iterations and an initial burn-in of 180,000. The final posterior probability of K, ln *p*(K), and Delta K (ΔK) was calculated using STRUCTURE HARVESTER^21^ to determine the most likely K value.

To assess the effect of the number of microsatellites on the stability of the genetic diversity and genetic structure, population genetic analysis was assessed by the following procedure: data files consisting of 5, 6, 7,…, 15, 20, 25, 30, 35, 40 microsatellite loci were created by resampling from the complete data set randomly using a python script and repeated 10 times for each subset of microsatellite loci. Genetic diversity parameters and genetic structure analysis were constructed for each replicated data set as described above. Statistical analysis was done used one sample *t*-test with the IBM-SPSS package version 24.

### Development of highly polymorphic microsatellite markers derived from SLAF sequences

Polymorphism is one of the important criteria for judging the usability of microsatellite markers. To determine if SLAF data of populations can be used for highly polymorphic microsatellite marker development, we selected 66 loci with both highly polymorphic microsatellite motif (at least 4 alleles/locus) and at most one individual missing data for primer design, and finally only 40 pairs of primers were synthesized since their flanking regions were long enough. All primers were designed by the program Primer v3 (http://bioinfo.ut.ee/primer3-0.4.0/). The primer size ranged from 18 to 22 bp with the optimal size of 20 bp. The optimum GC content was 50%, the optimum melting temperature was 60 °C (ranged from 50 to 65 °C), and the maximum acceptable difference between the melting temperatures of the forward and reverse primers was 5 °C.

### PCR validation and polymorphism examination

To test the use of polymorphic microsatellite markers we designed in *Rhododendron* species, total genomic DNA was extracted from 18 samples of population AES (*R. dauricum*), 10 samples of population JC (*R. mucronulatum*), moreover, and 12 individuals of *R. aureum* following a modified CTAB procedure^22^ and verified by electrophoresis on 1% agarose gel. PCR amplifications were performed in 20 μL reactions containing 50 ng genomic DNA, 1 × PCR buffer (plus Mg2+), 0.2 mM of dNTPs, and 0.5 μM of each primer, with each forward primer labeled with fluorescent dye (FAM, TAMRA, or HEX) (Invitrogen) and 1 unit (U) of Taq polymerase (Takara). Thermal cycling began with an initial denaturation step at 95 °C for 5 min, followed by 35 cycles of 30 s at 94 °C, 30 s at an optimal annealing temperature (Table 1), and 30 s at 72 °C, and a final elongation step at 72 °C for 8 min. The amplified fragments were resolved using an ABI 3730 DNA Analyzer (Applied Biosystems) using GeneScan 500 ROX as an internal size standard (Applied Biosystems, USA). Allele sizes were determined with the Peakscanner 2.0 software (Thermofisher Scientific, Germany).

**Table 1:** Characteristics of the 26 loci developed for *R. dauricum* and *R. mucronulatum*.

Locus	Primer sequences (5′-3′)	Ta (°C)	Motif	Size (bp)	*R. dauricum* (N = 18)			*R. mucronulatum* (N = 10)			*R. aureum* (N = 12)			Total (N = 40)	
					NA	Ar	Hs	NA	Ar	Hs	NA	Ar	Hs	NA	Ar
A	F: CAGAAGACAAGCCTCTAAAT	51	(AG)13	204–252	6	3.762	0.503	1	1	0	1	1	0	7	4.233
	R: GAGCCCAAAGATAACCAGTG														
B	F: AGCAGTTTTGGAGCCAG	52	(AC)11	200–212	4	3.228	0.458	5	4.397	0.728	3	2.515	0.255	7	3.855
	R: CTCAACATTTGCGAACACA														
C	F: AGCCCTAGTCAGCTTCGTGT	56	(TGG)6	240–273	4	3.525	0.557	3	2.682	0.361	4	3.86	0.664	9	5.39
	R: CCCAGAATTACCAAACCCTC														
D	F: ACCTTTCCAACTCCCTGCTT	52	(AG)11	350–378	5	4.582	0.791	2	1.995	0.306	9	7.798	0.909	15	7.673
	R: GTCGTCGTGTTTTCTCATTC														
E	F: CGTGCATCTCCTTCGTCTAC	55	(CA)10	152–182	8	5.699	0.788	4	3.682	0.667	6	5.14	0.759	13	7.568
	R: CAATCCGCCTATCAATCTGT														
G	F: TCTTCTTTGCTTCACAGT	45	(TC)19	212–260	9	7.195	0.892	8	6.762	0.861	8	6.948	0.873	19	9.339
	R: TATTTTCAGCCTTTTCCCAG														
H	F: CAATATACCAGCATGTCATC	48	(TTC)10	339–357	5	3.986	0.511	4	3.739	0.653	4	3.604	0.506	9	5.841
	R: CCTACAAGAGTTGGGAAATC														
K	F: CCTTGTTGCATCAGAATCGA	54	(AGAC)5	164–192	4	3.415	0.596	1	1	0	1	1	0	4	3.329
	R: TGACTAGGAGTGCTTCCACC														
O	F: CTTCTCCACCGTCTGGCTCG	54	(CT)8	323–335	5	3.586	0.472	2	1.778	0.111	3	2.963	0.582	6	4.014
	R: AGTTCTGTTGTTAGGGCTCC														
R	F: ACGTGATGAAAGCTGGTTAT	51	(TC)8	118–144	6	4.32	0.655	6	4.721	0.517	5	4.831	0.795	14	7.605
	R: GCTCGGGTTGTCTGGTTC														
X	F: GTGAAGAGGACGAGAAAGGA	45	(GA)9	232–252	6	5.186	0.822	7	7	0.929	6	5.097	0.767	11	7.188
	R: TCATGCCATATGTGCAAACG														
Y	F: ACCCTAACTTAACATCTTCG	45	(TC)10	250–278	2	1.98	0.324	3	2.982	0.594	3	2.869	0.473	8	5.635
	R: ACAAATGACATCAGCACTCT														
AD	F: ACCTGCGGTCTTCAGTGCTT	56	(CT)7	252–282	5	3.887	0.616	3	2.982	0.65	4	3.273	0.591	10	6.447
	R: GCCTTACAATATCCGTGTCG														
AE	F: AGAGGGAACCAACAATACGA	54	(AG)8	188–206	6	4.715	0.788	4	3.621	0.583	2	1.636	0.091	10	6.225
	R: ACGACCACCTGATGTGCGAC														
AF	F: GGTTTGGGTTTTGAGGAATG	54	(GA)11	189–229	4	2.952	0.381	1	1	0	8	6.509	0.777	12	5.003
	R: CTTGAAGCTGAGCTGAGTTA														
B1	F: GTTTTACAAGACCAGAGCGT	53	(AG)11	168–270	7	5.623	0.842	4	3.665	0.561	5	4.942	0.827	9	6.806
	R: CATTTCCGTTTCCTTCAGTC														
B8	F: TAAATGGCTAATCGTCCTAC	54	(CT)13	410–426	3	2.319	0.297	2	1.997	0.356	1	1	0	4	3.023
	R: AGCAAGTACAGCTCCGATGG														
B10	F: AGCCGACCCTTATGCATTAT	54	(CT)7	284–326	6	3.95	0.655	6	5.097	0.761	9	7.099	0.864	15	6.773
	R: CAAGCCCCCATCCTTTATCA														
B11	F: GGATCAAGAAGGTGGTCAAT	53	(GA)6	322–348	5	3.973	0.654	4	3.962	0.683	3	2.963	0.591	7	4.898
	R: CCAGTCAGCAATCAATAAGC														
B12	F: GCAAAATCATAACAAAAGCA	52	(CT)11	298–332	12	8.237	0.91	3	2.992	0.679	9	7.415	0.882	15	8.422
	R: AGCAGGAAACCCTATAAACA														
B13	F: GCGTTCAAAATCTCCAGAGC	56	(AG)7	318–340	7	5.317	0.814	7	6.608	0.839	3	2.921	0.594	12	6.599
	R: AGAACCCACTTGGATGCTGT														
B14	F: TTTAGAAAGGTCACTGACAC	47	(TG)7	270–306	6	5.218	0.781	6	5.524	0.806	5	4.236	0.695	13	7.182
	R: CTTGAAGTAATCGGCTATGT														
B16	F: AGTTGAGCAAGACAAGTGGA	54	(GA)8	266–290	8	5.167	0.773	2	2	0.489	8	6.973	0.886	13	7.341
	R: GCCTTCATTATGAAGTGGGT														
B17	F: GAGAAGGACGGGCATTTACC	56	(TC)8	218–238	3	2.509	0.304	4	3.679	0.6	4	3.564	0.514	8	5.247
	R: GGGCAGTTTTCCACTCATAC														
B19	F: ATCTGGAACAAACAGGACAT	52	(TTC)6	347–362	3	2.892	0.484	3	2.922	0.396	4	3.758	0.664	5	3.601
	R: CCATTATCGCTCTGAGTGTC														
B21	F: CGGTTCCATTTTCTGACTGG	56	(GA)7	148–202	11	7.935	0.917	6	5.32	0.811	12	9.745	0.968	19	8.904
	R: GTTTCTTTGGTTTTGGCTCT														

## Results

### Composition and characteristics of microsatellites in Rhododendron SLAF data

**Figure 1:**
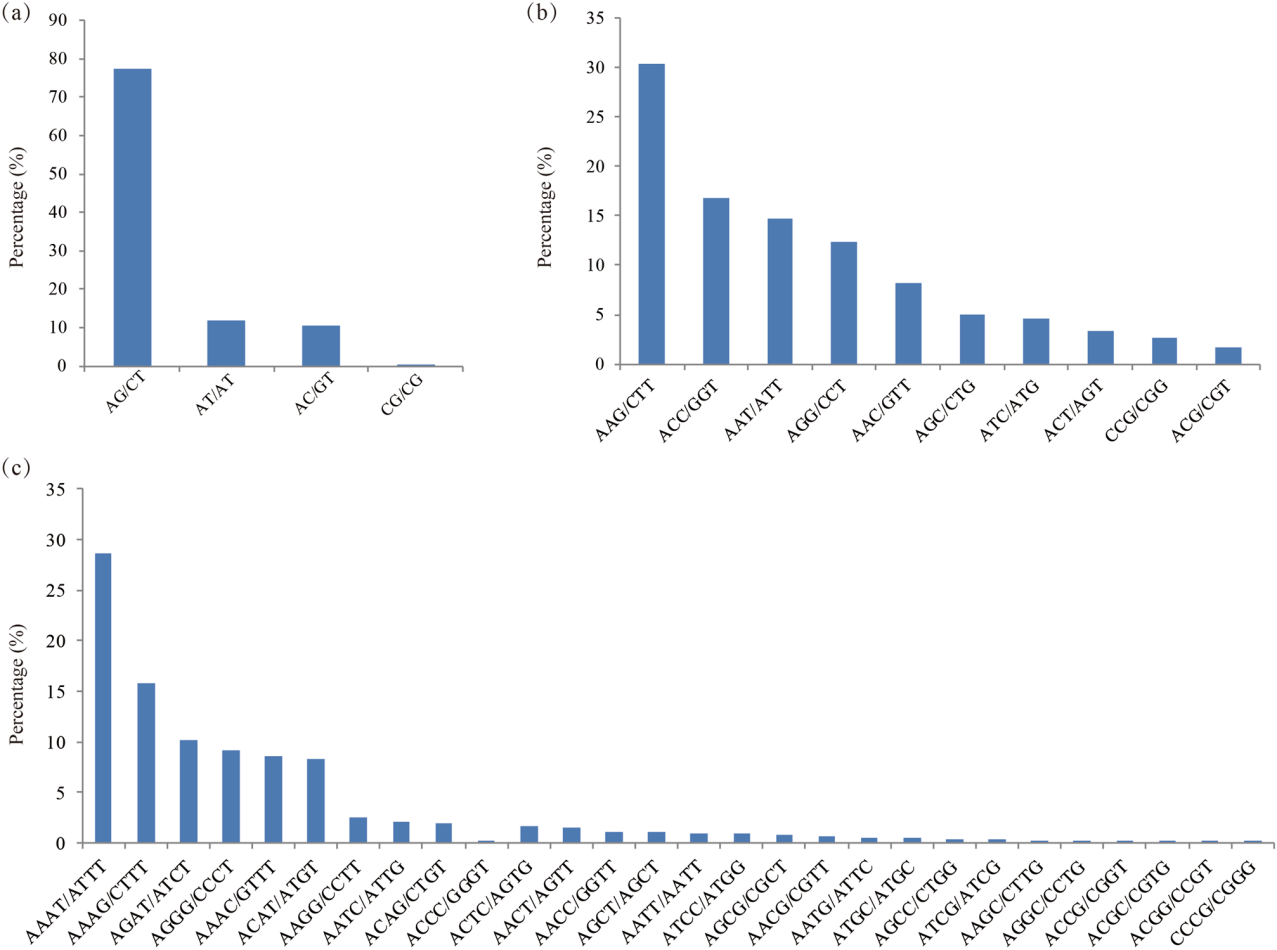
Frequency of consensus sequences containing di-nucleotide (**a**), tri-nucleotide (**b**) and tetra-nucleotides (**c**) motifs in *R. dauricum* and *R. mucronulatum*.

By analyzing the SLAF data of 63 individuals, we obtained 555,834 sequences. After screening these sequences, a total of 57,951 microsatellites were identified from 41,121 sequences and 10,705 sequences (26.03%) contained more than one microsatellite motif. Di-nucleotides were the most abundant repeat motif (86.10%, 49,584), followed by tri-nucleotides (11.69%), tetra-nucleotides (1.38), penta-nucleotides (0.48%) and hexa-nucleotides (0.34%). In addition, the highest number of repeats per locus was 6 (13,187, 22.90%) (Table 2). Of the di-nucleotides, the most frequent motifs were AG/CT repeats (38,339, 77.32%) (Fig. 1). The dominant repeats in the tri-nucleotide were AAG/CTT (2050, 30.44%), followed by ACC/GGT (1133, 16.82%) while ACG/CGT had the lowest frequency (117, 1.74%) Among the tetra-nucleotide motifs, AAAT/ATTT (227, 28.66%) was the most dominant motif (Fig. 1).

**Table 2:** The distribution of microsatellites based on the number of repeat units.

Repeat	Di	Tri	Tetra	Penta	Hexa	Total	Percentage (%)
5		3921	618	224	133	4896	8.50
6	11,430	1561	114	40	42	13,187	22.90
7	8384	619	42	15	12	9072	15.75
8	6803	305	12	1	4	7125	12.37
9	5501	141	5	0	3	5650	9.81
10	3997	77	1	0	2	4077	7.08
11	2730	39	0	0	3	2772	4.81
12	2108	21	0	0	0	2129	3.70
13	1747	23	0	0	0	1770	3.07
14	1421	11	0	0	0	1432	2.49
≥ 15	5463	17	0	0	1	5481	9.52
Total	49,584	6735	792	280	200	57,591	100
Percentage (%)	86.10	11.69	1.38	0.48	0.34	100

### Effects of the number of microsatellites on the stability of population genetics

Among the 41,121 sequences containing microsatellites, a total of 275 polymorphic microsatellites were selected after filtering by PLINK 2. The FIS values of ten loci deviated significantly from zero (p < 0.01), so they were excluded from subsequent analyses. Among the remaining 265 loci, the number of alleles per locus was 7, 6, 5, 4, 3 and 2 for 91, 48, 39, 18, 25 and 44 loci, respectively. For the remaining 265 loci, allelic richness (Ar) and the genetic diversity (Hs) measured 1.508 and 0.511 for *R. dauricum* and 1.445 and 0.444 for *R. mucronulatum*, respectively.

**Figure 2:**
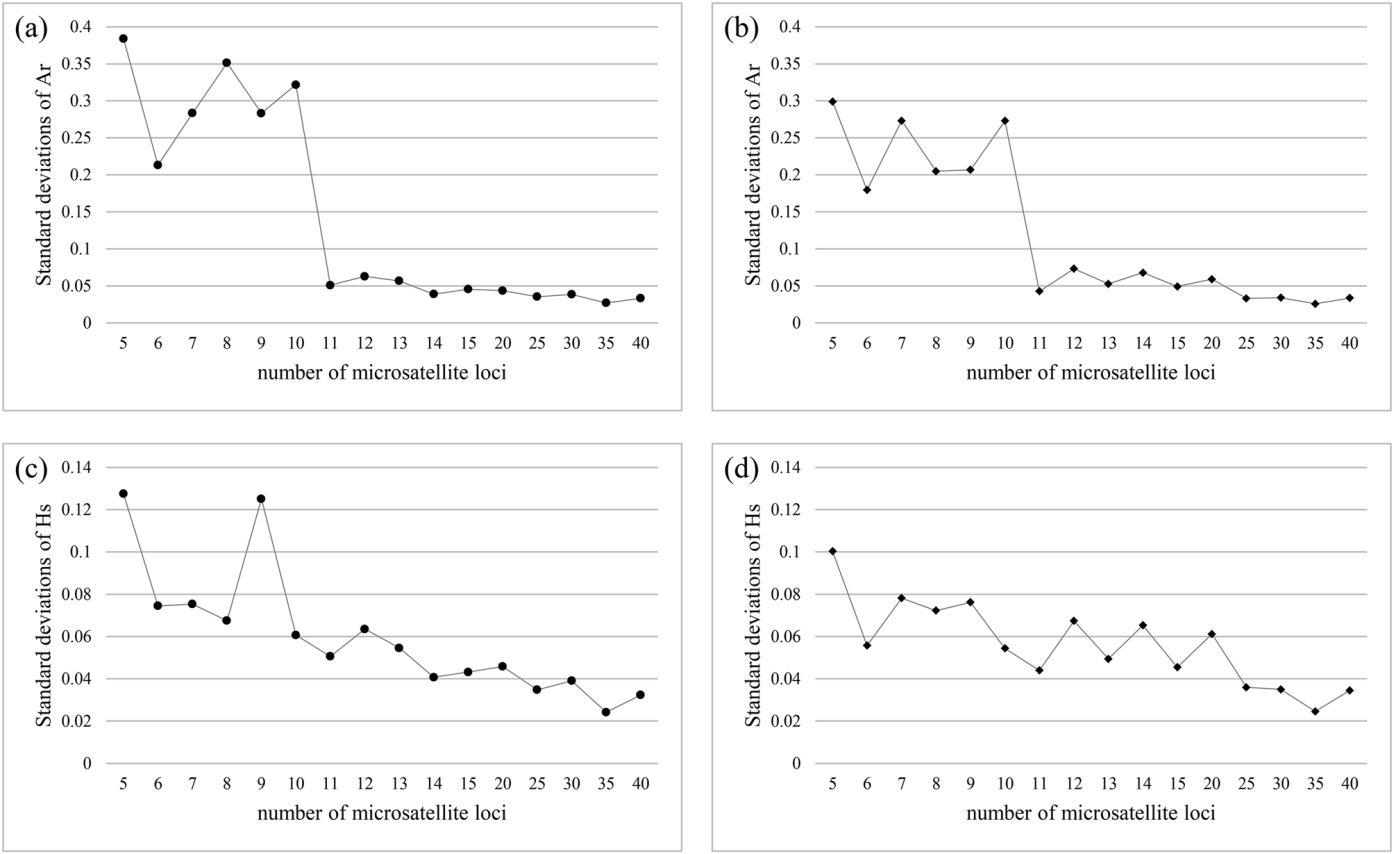
Standard deviation of allelic richness (Ar) and genetic diversity (Hs) per locus in *R. dauricum* (**a**,**c**) and *R. mucronulatum* (**b**,**d**), respectively.

Though the total mean values from all measurement repetitions did not deviate significantly from the value of 265 loci for any of the population genetic parameters (Fig. S1), the standard deviations of Ar and Hs decreased dramatically with increasing number of loci (Fig. 2). With five loci, the standard deviations of the Ar and Hs were very high, being up to 25% of the Ar and Hs based on all 265 loci (Fig. 2). Moreover, the absolute deviations were statistically significant when there were less than 11 microsatellites (p < 0.01). The average Ar for *R. dauricum* with 5 to 10 markers were from 1.553 to 1.745 (Fig. S1a), deviated 7.15% to 22.28% and a maximum of 44.16% to 67.04% from the number based on 265 loci (data not show). And for *R. mucronulatum*, the average Ar with 5 to 10 markers were from 1.483 to 1.630 (Fig. S1b), deviated 6.61% to 18.17% from the number based on 265 loci (data not show).

**Figure 3:**
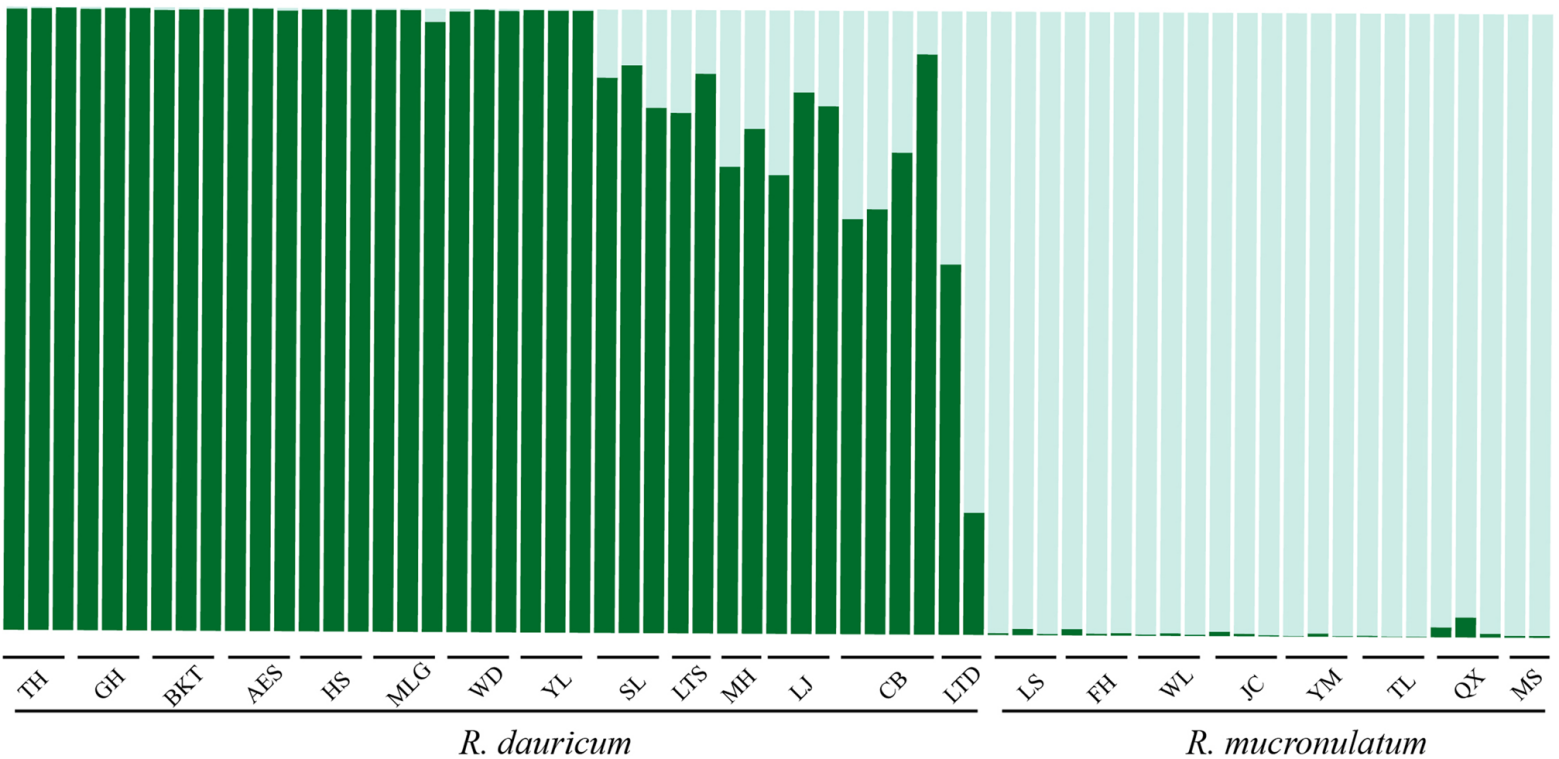
STRUCTURE results of the populations based 265 markers when K = 2. K = 2 was the most probable number of clusters.

**Figure 4:**
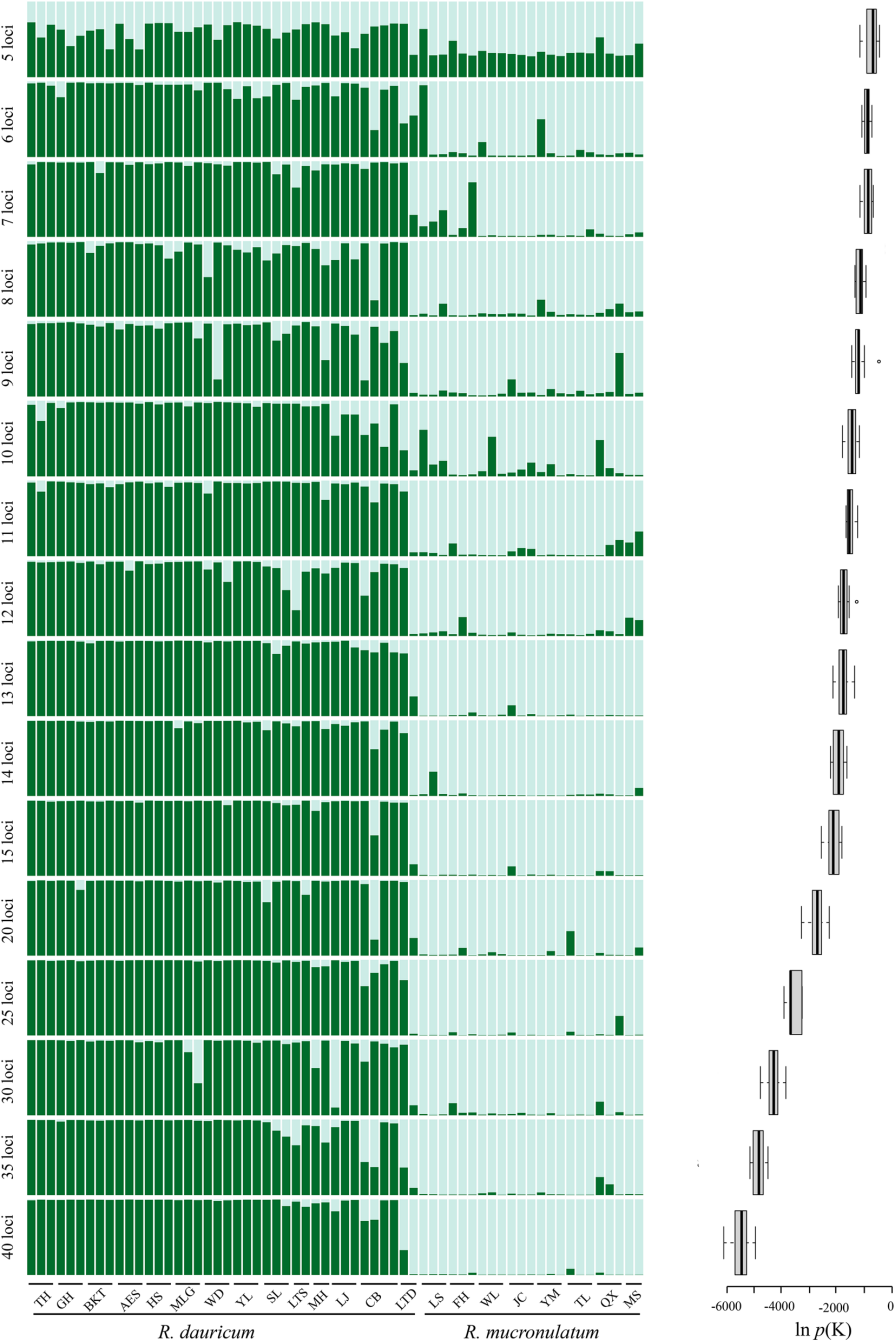
STRUCTURE results (left) and the mean Ln *p*(K) values (right) of different number of loci when K = 2 (left).

STRUCTURE analysis based all SSR markers (265 loci) showed clear differentiation between species (Fig. 3), similar to that detected by SNPs in our previous analysis^11^. All individuals were divided into two clusters according to the highest ΔK (Fig. S2). The resulting STRUCTURE plots for K = 2 of different microsatellite loci with the highest ln *p*(k) are given in Fig. 4. The number of admixed individuals decreased as more loci were used, especially in *R. mucronulatum*. Compared with the full set of 265 loci, with fewer than twelve loci, at least one incorrect cluster was detected in all datasets. Remarkably, when data of only five or six loci were used, the error rate of populations clustering reached 50%.

### PCR validation of SSR primers

Among the 265 polymorphic microsatellites, a total of 40 primer pairs were designed for evaluating PCR amplification efficiency and polymorphism in congeneric species. Of the primers tested, 14 primers were excluded from further analysis because these primers did not generate clear microsatellite peaks or failed to be amplified. The remaining 26 primers exhibited high amplification success and were screened in 40 samples from three species. Of the 26 polymorphic SSR loci, 23 were dinucleotides, two were trinucleotides and one was a tetranucleotide.

In total, 274 alleles were detected across all individuals, with the number of alleles per locus (NA) ranging from 4 to 19. The Ar values ranged from 3.023 to 9.339, with an average of 6.082, while the HS varied from 0.297 to 0.917, 0 to 0.929 and 0 to 0.968 in *R. dauricum*, *R. mucronulatum* and *R. aureum*, respectively (Table 1).

## Discussion

Simple sequence repeats (SSRs) are co-dominant nuclear markers that are widely used in population genetic studies, which provide insights and guidelines for preserving the genetic diversity of populations^23,24^. Large number of papers published in the last few years have involved the use of microsatellites. However, as shown here and elsewhere, the low number of loci used may lead to erroneous conclusions when comparing populations^3^.

For example, in our study, significant deviations in Ar were found when using less than 11 loci, compared to the value from the full dataset of 265 loci. Moreover, the genetic diversity parameter Hs of *R. dauricum* was significantly different from the Hs based 265 loci (0.511) when compared to analyses of less than 10 microsatellites (Fig. 2).While for *R. mucronulatum*, the Hs value already deviated significantly from the Hs based 265 loci (0.444) when used 25 microsatellite loci (Fig. 2), it may be caused by few individuals of *R. mucronulatum* in this study. Experimental studies in red deer showed that significant deviations from the actual values for sample sizes of less than 30 per population^4^. Allelic richness (Ar), one of the most reported measures of genetic variation, is also referred to as allelic diversity or mean number of alleles per locus. And in FSTAT software, the Ar is corrected for sample size, thus compared with Ar, Hs is more sensitive to the sample size.

These effects could also be confirmed for the population genetic structure, which was investigated by STRUCTURE. Arthofer et al. demonstrate that the population structure was still retained, though about a quarter of a individuals cannot be correctly assigned, when using only two loci with the Arwere 12.94 and 13.54, , respectively^5^, which were much larger than the average Ar of microsatellite sites in the other study^25–27^. Thus, it is difficult to de novo develop microsatellite primers with such high polymorphism. Therefore, two microsatellite loci are far from enough in actual genetic structure research. Moreover, our previous study illustrated *R. dauricum* and *R. mucronulatum* clustered into distinct groups and showed majority populations collected from the Changbai Mountains (MES, SL, LTS, MH, HC, LJ, CB, WT) of *R. dauricum* with some admixture from *R. mucronulatum*^11^. However, in our study, the differentiation between species was not obvious with low microsatellite loci used. Remarkably, the incorrect assignment was still possible with fewer than 12 loci. With decreasing number of microsatellites, a reliable comparison between species cannot be achieved, even if Bayesian methods are used.

In addition, 26 polymorphic microsatellite loci were validated and characterized for individuals of *R. dauricum*, *R. mucronulatum* and *R. aureum*. The levels of diversity observed at these microsatellite loci, measured as allelic richness (Ar) and genetic diversity (Hs), were similar to those in previous studies^11^. *Rhododendron* is a familiar ornamental plant worldwide, ranging from tropical to polar climates^13^. This study provides a potentially highly polymorphic SSR markers library for the research of *Rhododendron* subgen. *Rhodorastrum*, which will facilitate the further study of the genetics of *Rhododendron* subgen. *Rhodorastrum*, even *Rhododendron*. Furthermore, we explored a simple route to develop polymorphic SSR markers from non-model species based on SLAF-seq, which is well suited for polymorphic SSR marker discovery in non-model organisms.

## Conclusion

Previous studies showed considerable differences of genetic diversity and genetic structure with regard to the number of microsatellite loci. Our results indicated significant effects on population genetic parameters if the number of microsatellite loci was less than 12. With decreasing marker numbers, the accuracy of population genetic of and the genetic structure decreases. Fortunately, the SLAF-seq data of populations offers an effective approach to develop polymorphic microsatellite markers for non-model species. The 26 polymorphic microsatellite markers we developed for *Rhododendron* species will be important for investigating population genetic diversity and genetic structure, and these results in turn will provide crucial information for conservation and management of *Rhododendron* species.

## Supplementary Information

Supplementary Information.
