# Supplementary material for: Impact of different numbers of microsatellite markers on population genetic results using SLAF-seq data for Rhododendron species
Source: Sci Rep. 2021 Apr 21;11:8597. doi: 10.1038/s41598-021-87945-x (PMC8060317; doi:10.1038/s41598-021-87945-x)
Supplement: Supplementary file 1 — Supplementary Information. [file 41598_2021_87945_MOESM1_ESM.docx]

Impact of different numbers of microsatellite markers on population genetic results using SLAF-seq data for *Rhododendron* species

Huaying Wang^a^, Baiming Yang^a^, Huan Wang^a^, Hongxing Xiao^a^*

^a^Key Laboratory of Molecular Epigenetics of Ministry of Education, Northeast Normal University, Changchun 130024, China.

Authors:

Hua-Ying Wang: wanghy609@nenu.edu.cn

Bai-Ming Yang: yangbm660@nenu.edu.cn

Huan Wang: wangh624@nenu.edu.cn

***Corresponding author at:** Hong-Xing Xiao, Key Laboratory of Molecular Epigenetics of Ministry of Education, Northeast Normal University, Changchun 130024, China.

*E-mail address***:** xiaohx771@nenu.edu.cn

Running title: Impact of different numbers of microsatellite markers


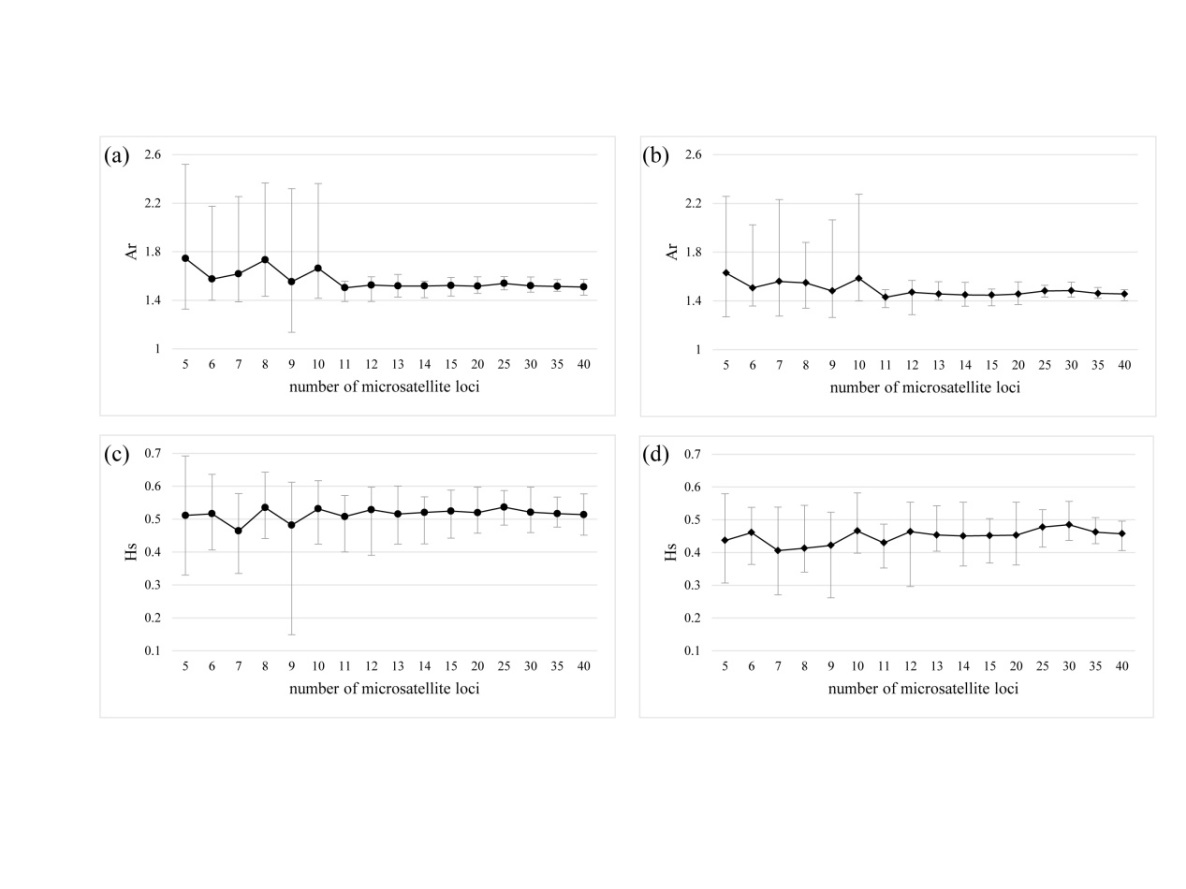


Figure S1 The average value of allelic richness (Ar) and genetic diversity (Hs) per locus in *R. dauricum* (a,c) and *R. mucronulatum* (b,d), respectively.


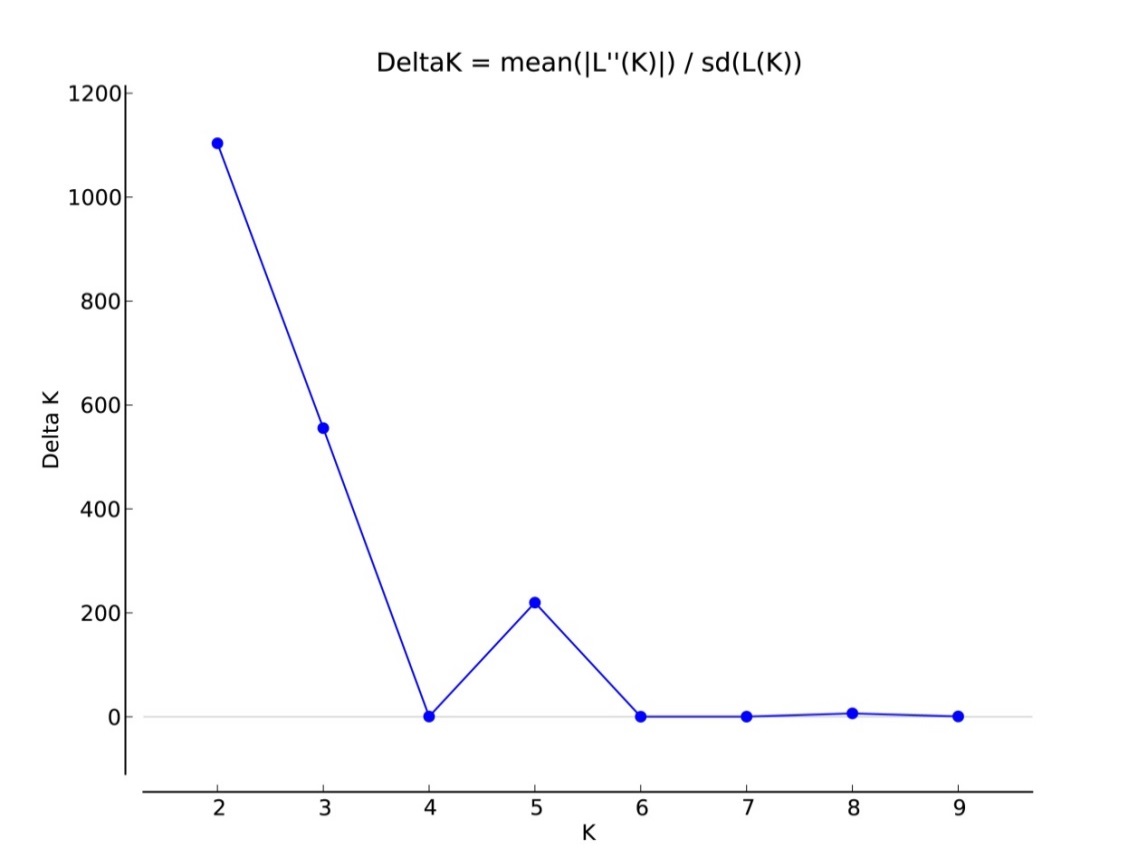


Figure S2 ΔK from genetic structure analysis of the two species *R. dauricum* and *R. mucronulatum* based on 265 loci.

Table S1 Sampling information of SLAF data of *R. dauricum* and *R. mucronulatum* in our previous study (size1) and this study (size2).

| pop | Location | Long (°E) | Lat (°N) | voucher | Size1 | Size2 |
| --- | --- | --- | --- | --- | --- | --- |
| ***R. dauricum*** |  |  |  |  | 40 | 38 |
| AES+ | Aersha, IM | 120.562 | 47.410 | NENU00042001 | 2 | 3 |
| TP+ | Taipingling, IM | 120.491 | 47.488 | NENU00042002 | 1 | - |
| BKT | Boktu, IM | 122.105 | 48.832 | NENU00042004 | 3 | 3 |
| GH* | Genhe City, IM | 121.492 | 50.775 | NENU00042005 | 1 | 3 |
| JH* | Jinhe Town, IM | 121.294 | 51.041 | NENU00042006 | 1 | - |
| AK* | Oakley Hill, IM | 122.037 | 51.838 | NENU00042007 | 1 | - |
| TH | Tahe County, HLJ | 124.710 | 52.334 | NENU00042009 | 3 | 3 |
| HS | Big black mountain, HLJ | 126.448 | 50.241 | NENU00042010 | 3 | 3 |
| MLG | Maolangou, HLJ | 129.771 | 49.098 | NENU00042012 | 3 | 3 |
| WD | Longmen Shizhai, HLJ | 126.335 | 48.695 | NENU00042013 | 3 | 3 |
| YL | Yilan County, HLJ | 129.568 | 46.325 | NENU00042014 | 3 | 3 |
| MES | Maoer Mountain, HLJ | 127.449 | 45.259 | NENU00042015 | 1 | 0 |
| SL | Shulan City, JL | 127.452 | 44.247 | NENU00042016 | 3 | 3 |
| LTS | Camel Mountain, JL | 129.540 | 43.645 | NENU00042019 | 2 | 2 |
| MH | Manhe Village, JL | 130.055 | 43.189 | NENU00042020 | 2 | 2 |
| HC | Jiushaping, JL | 130.633 | 42.417 | NENU00042021 | 1 | 0 |
| CB# | Changbai Mountain, JL | 127.787 | 42.052 | NENU00042022 | 3 | 4 |
| WT# | Wangtian'e Scenic Area, JL | 127.943 | 41.547 | NENU00042024 | 1 | - |
| LJ | Pearl Gate Village, JL | 126.156 | 42.278 | NENU00042026 | 3 | 3 |
| ***R. mucronulatum*** | |  |  |  | 25 | 25 |
| LTD | Lao Baldingzi, LN | 124.908 | 41.298 | NENU00042028 | 2 | 2 |
| LS | Sitaizi Forest Farm, JL | 124.702 | 43.189 | NENU00042029 | 3 | 3 |
| FH | Phoenix Mountain, LN | 124.078 | 40.413 | NENU00042031 | 3 | 3 |
| WL | Wulong Mountain, LN | 124.336 | 40.255 | NENU00042032 | 3 | 3 |
| JC | Sandaogou, LN | 119.345 | 40.583 | NENU00042035 | 3 | 3 |
| YM | Yunmeng Mountain, BJ | 116.686 | 40.555 | NENU00042036 | 3 | 3 |
| TL | Tuoliang Scenic Spot,HB | 113.812 | 38.740 | NENU00042037 | 3 | 3 |
| QX | Leshan Park, SD | 121.066 | 37.226 | NENU00042038 | 3 | 3 |
| MS | Mengshan Forest Park, SD | 117.969 | 35.557 | NENU00042039 | 2 | 2 |
| Total |  |  |  |  | 65 | 63 |

IM: Inner Mongolia; HLJ: Heilongjiang Province; JL: Jilin Province; LN: Liaoning Province; BJ: Beijing; HB: Hebei Province; SD: Shandong Province. “-” represents the merged populations, and population name with the same mark on the right are merged into one population (+; *; #).
